# Supplementary material for: Impacts of the COVID-19 pandemic on deprivation-level differences in cardiovascular hospitalisations: a comparison of England and Denmark using the OpenSAFELY platform and National Registry Data
Source: BMJ Open. 2024 Oct 15;14(10):e088710. doi: 10.1136/bmjopen-2024-088710 (PMC11481132; doi:10.1136/bmjopen-2024-088710)

Supplementary materials

Table of contents

Figure S1: COVID-19 stringency index and confirmed COVID-19 deaths in the United Kingdom and Denmark 2

Table S1: Danish income quintile thresholds by age 3

Information governance and ethical approval 6

Table S2: Characteristics of English and Danish cohorts as of 1st March 2019 7

Table S3: Characteristics of English and Danish cohorts as of 1st March 2021 9

Table S4 Estimated number of events during the pre-pandemic period (May 2018-February 2020) and during the pandemic period (March 2020-December 2021) with and without COVID-19 restrictions in England. 11

Table S5 Estimated number of events during the pre-pandemic period (May 2018-February 2020) and during the pandemic period (March 2020-December 2021) with and without COVID-19 restrictions in Denmark. **14**

Figure S2: Monthly change (first derivative) in percentage of population with hospital admissions for each outcome in England 17

Figure S3: Monthly change (first derivative) in percentage of population with hospital admissions for each outcome in Denmark 18

Figure S1: COVID-19 stringency index and confirmed COVID-19 deaths in the United Kingdom and Denmark

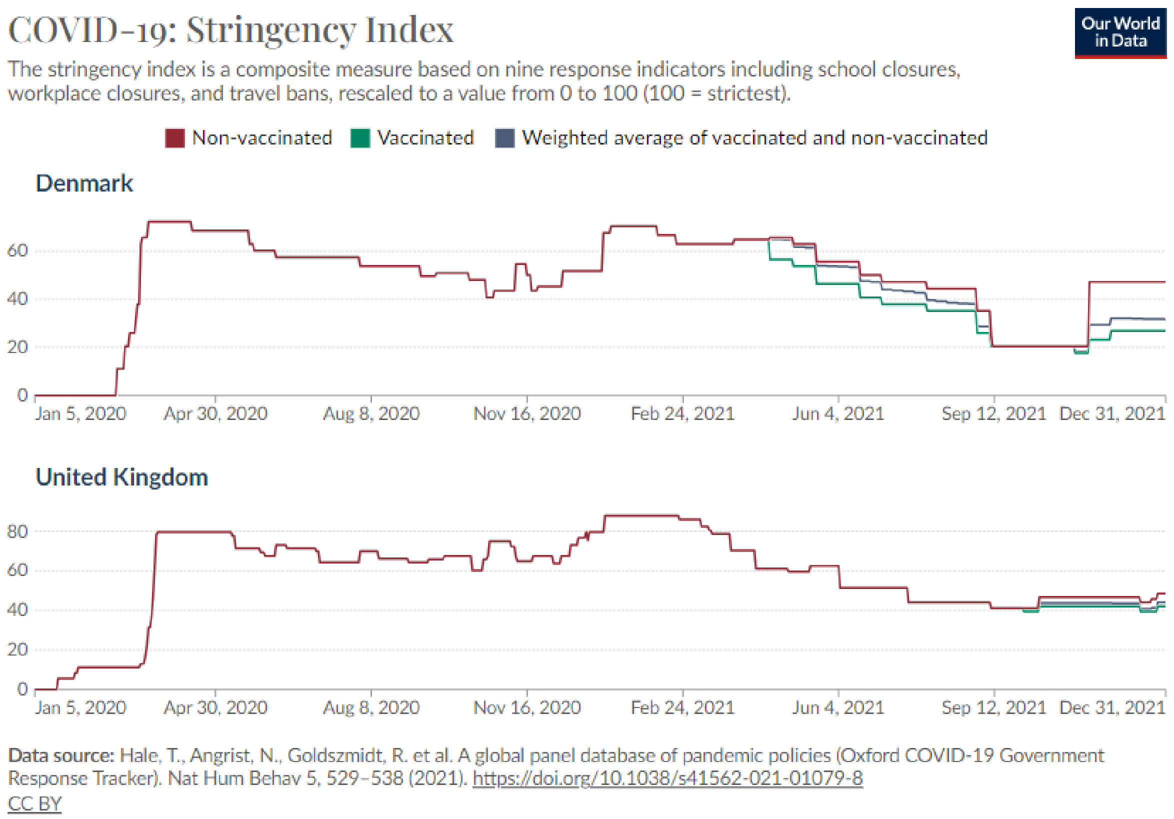

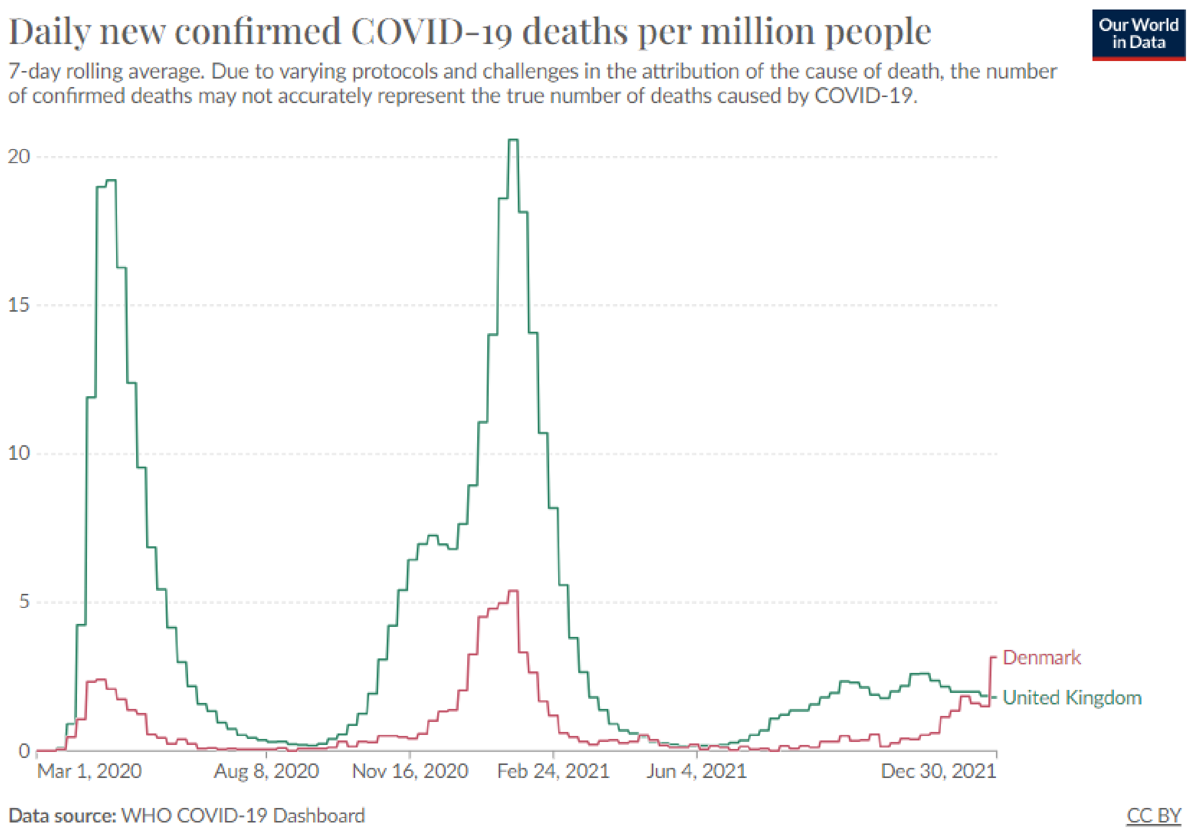

Table S1: Danish income quintile thresholds by age

Age	p20_EUR	p40_EUR	p60_EUR	p80_EUR
18	25182	72916	113666	150954
19	16410	45070	93213	141443
20	13808	26629	52777	111849
21	13584	23915	39847	75574
22	14709	24972	40259	69040
23	16221	27728	43680	70735
24	16392	28706	43212	66016

25	19506	34540	51115	75810
26	22470	40495	59474	84861
27	27020	45627	66811	91558
28	30462	50242	73609	97650
29	33471	54836	80029	103119
30	36405	59234	84719	107529
31	38774	63156	88893	111923
32	40835	66083	92045	115296
33	42702	68970	94971	119104
34	44419	71603	97645	122261
35	45197	73873	99855	125086
36	46870	75894	101873	128082
37	48078	78599	104282	131261
38	48998	79903	105971	133703
39	50160	81780	107947	136459
40	50379	82324	109129	138591
41	51080	83139	109933	139837
42	51717	84015	111295	142179
43	51668	84826	112578	144983

44	51499	84458	112709	146030
45	51923	85140	113956	147824
46	51986	85092	114176	148753
47	51048	84006	113080	148934
48	50309	83096	112877	148588
49	50456	83042	112546	148340
50	50419	82667	111829	148155
51	50217	82488	110889	146641
52	49285	80909	109046	144300
53	48459	79177	106941	141183
54	48745	78956	106260	139949
55	47567	76712	103671	136536
56	47100	75951	102339	133952
57	45956	73882	100135	131312
58	46055	72543	98054	128610
59	45267	70752	95914	125318
60	44704	68828	93708	123101
61	44527	66792	90723	119574
62	41205	60450	82624	112747

63	38763	57002	76277	105089
64	37439	53745	71035	98313
65	36607	49956	65004	91186
66	35667	48443	62382	85981
67	34423	46849	59731	81559
68	34403	45971	58114	79102
69	34222	45431	56805	76917
70	33622	44319	55098	74172
71	33375	43675	53797	72148
72	32626	42808	52307	70001
73	32027	41881	50661	67358
74	31353	40825	49147	66004
75	30837	39814	47483	64164
76	30303	38894	45992	61614
77	29797	37666	44750	59413
78	29559	37001	43971	58162
79	29370	36193	43190	56651
80	28757	35022	42021	54662
81	28385	34129	40959	52711

82	27844	32924	39839	50633
83	27550	32167	39079	49887
84	27280	31707	38525	48224
85	26758	31061	37573	46637
86	26684	30875	37046	45868
87	26416	30562	36288	45061
88	26201	30239	35620	44415
89	26352	30046	35183	43689
90	26066	29829	34498	42868
91	26109	29693	33904	42409
92	25861	29545	33560	41600
93	25777	29471	33491	41447
94	25748	29217	32653	40505
95	25787	29125	32621	40789
96	25500	29017	32155	39489
97	25182	29109	32050	38953
98	26479	29237	32696	40218
99	26264	29587	32396	40124
100	25613	28769	32105	38579

Information governance and ethical approval

Patient data has been pseudonymised for analysis and linkage using industry standard cryptographic hashing techniques; all pseudonymised datasets transmitted for linkage onto OpenSAFELY are encrypted; access to the NHS England OpenSAFELY COVID-19 service is via a virtual private network (VPN) connection; the researchers hold contracts with NHS England and only access the platform to initiate database queries and statistical models; all database activity is logged; only aggregate statistical outputs leave the platform environment following best practice for anonymisation of results such as statistical disclosure control for low cell counts [1]

The service adheres to the obligations of the UK General Data Protection Regulation (UK GDPR) and the Data Protection Act 2018. The service previously operated under notices initially issued in February 2020 by the the Secretary of State under Regulation 3(4) of the Health Service (Control of Patient Information) Regulations 2002 (COPI Regulations), which required organisations to process confidential patient information for COVID-19 purposes; this set aside the requirement for patient consent [2]. As of 1 July 2023, the Secretary of State has requested that NHS England continue to operate the Service under the COVID-19 Directions 2020 [3]. In some cases of data sharing, the common law duty of confidence is met using, for example, patient consent or support from the Health Research Authority Confidentiality Advisory Group [4].

Taken together, these provide the legal bases to link patient datasets using the service. GP practices, which provide access to the primary care data, are required to share relevant health information to support the public health response to the pandemic, and have been informed of how the service operates.

[1] ISB1523: Anonymisation Standard for Publishing Health and Social Care Data. NHS Digit n.d. https://digital.nhs.uk/data-and-information/information-standards/information-standards-and-data-collections-including-extractions/publications-and-notifications/standards-and-collections/isb1523-anonymisation-standard-for-publishing-health-and-social-care-data (accessed September 20, 2023).

[2] [Withdrawn] [withdrawn] Coronavirus (COVID-19): notice under regulation 3(4) of the Health Service (Control of Patient Information) Regulations 2002 – general. GOVUK 2022. https://www.gov.uk/government/publications/coronavirus-covid-19-notification-of-data-controllers-to-share-information/coronavirus-covid-19-notice-under-regulation-34-of-the-health-service-control-of-patient-information-regulations-2002-general--2 (accessed September 20, 2023).

[3] COVID-19 Public Health Directions 2020. NHS Digit n.d. https://digital.nhs.uk/about-nhs-digital/corporate-information-and-documents/directions-and-data-provision-notices/secretary-of-state-directions/covid-19-public-health-directions-2020 (accessed September 20, 2023).

[4] Confidentiality Advisory Group. Health Res Auth n.d. https://www.hra.nhs.uk/about-us/committees-and-services/confidentiality-advisory-group/ (accessed September 20, 2023).

Table S2: Characteristics of English and Danish cohorts as of 1st March 2019

Characteristic		England* N=15,623,860 n (%)	Denmark N= 4514317 n (%)
Age category	18 - 40 years	5,561,685 (35.6)	1,557,900 (34.5)
	41 - 60 years	5,222,525 (33.4)	1,547,804 (34.3)
	61 - 80 years	3,918,280 (25.1)	1,179,017 (26.1)
	>80 years	921,370 (5.9)	229,596 (5.1)
Sex	Female	7,913,915 (50.7)	2,293,371 (50.8)
	Male	7,709,950 (49.3)	2,220,946 (49.2)
Deprivation*	1 (Most deprived)	3,064,460 (19.6)	885,179 (19.6)
	2	3,114,535 (19.9)	905,664 (20.1)
	3	3,387,880 (21.7)	907,145 (20.1)
	4	3,169,585 (20.3)	908,171 (20.1)
	5 (Least deprived)	2,887,405 (18.5)	908,158 (20.1)
Rural-Urban	Rural	3,376,165 (21.6)	-
	Urban	12,247,700 (78.4)	-

Diabetes		1,201,440 (7.7)	309,468 (6.9)
Asthma		1,351,940 (8.7)	627,288 (13.9)
COPD		486,310 (3.1)	440,972 (9.8)

*England data is rounded to the nearest 5.

Table S3: Characteristics of English and Danish cohorts as of 1st March 2021

Characteristic		England* N=16,139,075 n (%)	Denmark N= 4358665 n (%)
Age category	18 - 40 years	5,636,175 (34.9)	1,380,568 (31.7)
	41 - 60 years	5,333,495 (33)	1,529,705 35.1
	61 - 80 years	4,126,510 (25.6)	1,202,524 (27.6)
	>80 years	1,042,895 (6.5)	245,868 (5.6)
Sex	Female	8,157,995 (50.5)	2,218,928 (50.9)
	Male	7,981,085 (49.5)	2,139,737 (49.1)
Deprivation*	1 (Most deprived)	3,176,660 (19.7)	830,850 (19.1)
	2	3,225,975 (20)	872,689 (20.0)
	3	3,499,040 (21.7)	880,638 (20.2)
	4	3,266,395 (20.2)	886,462 (20.3)

	5 (Least deprived)	2,971,010 (18.4)	888,026 (20.4)
Rural-Urban	Rural	3,467,460 (21.5)	-
	Urban	12,671,620 (78.5)	-
Diabetes		1,316,685 (8.2)	330,801 (7.6)
Asthma		1,426,670 (8.8)	634,776 (14.6)
COPD		516,940 (3.2)	453,375 (10.4)

*England data is rounded to the nearest 5.

Table S4 Estimated number of events during the pre-pandemic period (May 2018-February 2020) and during the pandemic period (March 2020-December 2021) with and without COVID-19 restrictions in England.

Outcome	Deprivation quintile	Estimated number of events pre-pandemic	Estimated number of events during pandemic period if pre-pandemic trends continued	Estimated number of events during pandemic with COVID-19 restrictions	Difference in estimated events with and without COVID-19 restrictions	% difference in estimated events with and without COVID-19 restrictions
		n (95% confidence interval)				% (95% confidence interval)
Heart failure	1 (Most deprived)	8,512<br>(8,090 - 8,955)	14,636<br>(13,514 - 15,853)	12,028<br>(11,504 - 12,576)	-2608	-17.8
	2	8,168<br>(7,866 - 8,482)	14,035<br>(13,062 - 15,084)	11,832<br>(11,441 - 12,236)	-2203	-15.7
	3	8,378<br>(8,104 - 8,662)	14,375<br>(13,408 - 15,414)	12,431<br>(12,069 - 12,803)	-1944	-13.5
	4	7,398<br>(7,104 - 7,704)	12,671<br>(11,773 - 13,641)	11,240<br>(10,853 - 11,641)	-1431	-11.3

	5 (Least deprived)	6,355 (6,016 - 6,714)	10,878 (10,015 - 11,818)	9,899 (9,447 - 10,373)	-979	-9
Myocardial infarction	1	9,683 (9,420 - 9,954)	11,859 (11,340 - 12,402)	10,751 (10,463 - 11,046)	-1108	-9.3
	2	9,421 (9,229 - 9,617)	11,536 (11,077 - 12,016)	10,507 (10,296 - 10,723)	-1029	-8.9
	3	9,802 (9,626 - 9,982)	11,980 (11,516 - 12,462)	10,962 (10,769 - 11,160)	-1018	-8.5
	4	8,779 (8,590 - 8,973)	10,707 (10,273 - 11,159)	9,844 (9,635 - 10,056)	-863	-8.1
	5	7,651 (7,430 - 7,878)	9,320 (8,902 - 9,759)	8,609 (8,365 - 8,860)	-711	-7.6

Stroke	1	8,895<br>(8,661 - 9,135)	11,697<br>(11,222 - 12,192)	12,316<br>(12,026 - 12,613)	619	5.3
	2	9,068<br>(8,890 - 9,249)	11,921<br>(11,485 - 12,373)	12,580<br>(12,357 - 12,807)	659	5.5
	3	9,884<br>(9,719 - 10,053)	12,970<br>(12,515 - 13,443)	13,719<br>(13,509 - 13,932)	749	5.8
	4	9,275<br>(9,092 - 9,461)	12,145<br>(11,700 - 12,608)	12,876<br>(12,646 - 13,109)	731	6
	5	8,467<br>(8,243 - 8,698)	11,078<br>(10,626 - 11,549)	11,770 (11,491 - 12,056)	692	6.2
Venous thromboembolism	1	6,272<br>(6,046 - 6,507)	8,549<br>(8,063 - 9,065)	7,703 (7,441 - 7,974)	-846	-9.9
	2	6,248<br>(6,080 - 6,422)	8,514<br>(8,075 - 8,977)	7,687 (7,490 - 7,889)	-827	-9.7

	3	6,656 (6,499 - 6,816)	9,052 (8,602 - 9,528)	8,190 (8,007 - 8,377)	-862	-9.5
	4	6,103 (5,933 - 6,278)	8,284 (7,853 - 8,740)	7,510 (7,312 - 7,714)	-774	-9.3
	5	5,444 (5,241 - 5,656)	7,383 (6,957 - 7,836)	6,707 (6,471 - 6,952)	-676	-9.2

Table S5 Estimated number of events during the pre-pandemic period (May 2018-February 2020) and during the pandemic period (March 2020-December 2021) with and without COVID-19 restrictions in Denmark.

Outcome	Deprivation quintile	Estimated number of events pre-pandemic	Estimated number of events during pandemic period if pre-pandemic trends continued	Estimated number of events during pandemic with COVID-19 restrictions	Difference in estimated events with and without COVID-19 restrictions	% difference in estimated events with and without COVID-19 restrictions
		n (95% confidence interval)				

Heart failure	1	2,434 (2,327 - 2,546)	2,176 (2,013 - 2,352)	2,240 (2,131 - 2,355)	64	2.9
	2	2,249 (2,174 - 2,327)	2,045 (1,903 - 2,198)	2,110 (2,033 - 2,190)	65	3.2
	3	2,021 (1,960 - 2,085)	1,849 (1,723 - 1,984)	1,912 (1,849 - 1,977)	63	3.4
	4	1,815 (1,747 - 1,885)	1,668 (1,549 - 1,796)	1,728 (1,659 - 1,801)	60	3.6
	5	1,627 (1,546 - 1,711)	1,497 (1,380 - 1,624)	1,555 (1,471 - 1,644)	58	3.9
Myocardial infarction	1	3,660 (3,516 - 3,810)	4,626 (4,315 - 4,961)	3,613 (3,462 - 3,770)	-1013	-21.9
	2	3,200 (3,104 - 3,299)	4,116 (3,859 - 4,390)	3,190 (3,089 - 3,293)	-926	-22.5

	3	2,721 (2,645 - 2,801)	3,521 (3,304 - 3,752)	2,708 (2,628 - 2,790)	-813	-23.1
	4	2,312 (2,231 - 2,396)	3,005 (2,810 - 3,214)	2,293 (2,208 - 2,382)	-712	-23.7
	5	1,961 (1,869 - 2,058)	2,552 (2,368 - 2,750)	1,933 (1,836 - 2,035)	-619	-24.3
Stroke	1	3,409 (3,295 - 3,527)	3,433 (3,239 - 3,639)	3,097 (2,983 - 3,215)	-336	-9.8
	2	3,170 (3,090 - 3,253)	3,248 (3,078 - 3,427)	3,007 (2,924 - 3,092)	-241	-7.4
	3	2,867 (2,801 - 2,935)	2,955 (2,804 - 3,114)	2,807 (2,739 - 2,877)	-148	-5
	4	2,590 (2,518 - 2,665)	2,682 (2,539 - 2,834)	2,615 (2,538 - 2,694)	-67	-2.5

	5	2,337 (2,250 - 2,427)	2,423 (2,280 - 2,575)	2,424 (2,328 - 2,524)	1	0
Venous thromboembolism	1	3,932 (3,811 - 4,056)	4,606 (4,372 - 4,853)	4,224 (4,092 - 4,361)	-382	-8.3
	2	3,688 (3,603 - 3,776)	4,396 (4,191 - 4,612)	4,076 (3,980 - 4,174)	-320	-7.3
	3	3,365 (3,295 - 3,437)	4,034 (3,850 - 4,228)	3,782 (3,703 - 3,863)	-252	-6.2
	4	3,067 (2,989 - 3,147)	3,694 (3,517 - 3,880)	3,501 (3,413 - 3,592)	-193	-5.2
	5	2,791 (2,696 - 2,888)	3,366 (3,188 - 3,554)	3,226 (3,116 - 3,339)	-140	-4.2

Figure S2: Monthly change (first derivative) in percentage of population with hospital admissions for each outcome in England

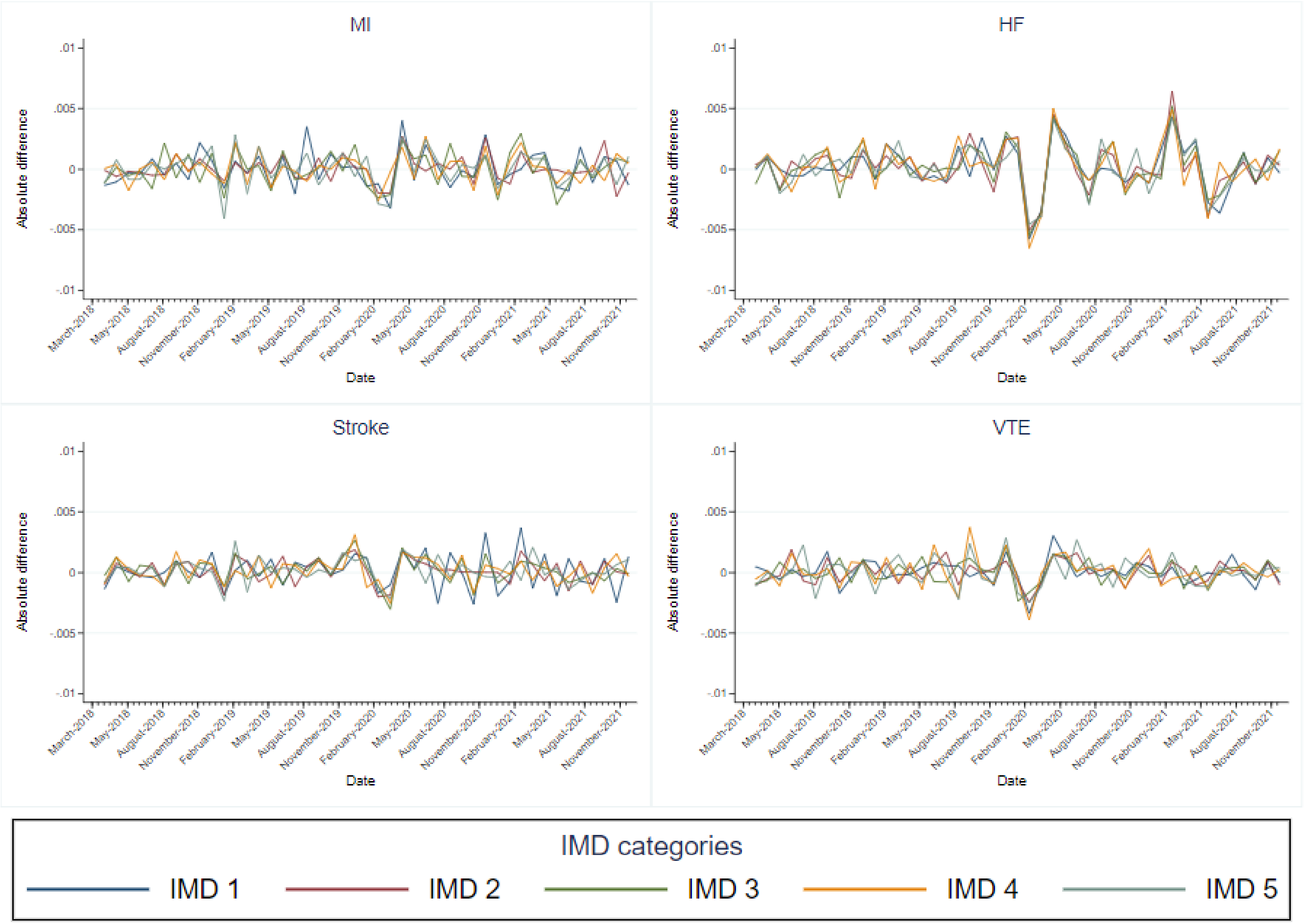

Figure S3: Monthly change (first derivative) in percentage of population with hospital admissions for each outcome in Denmark

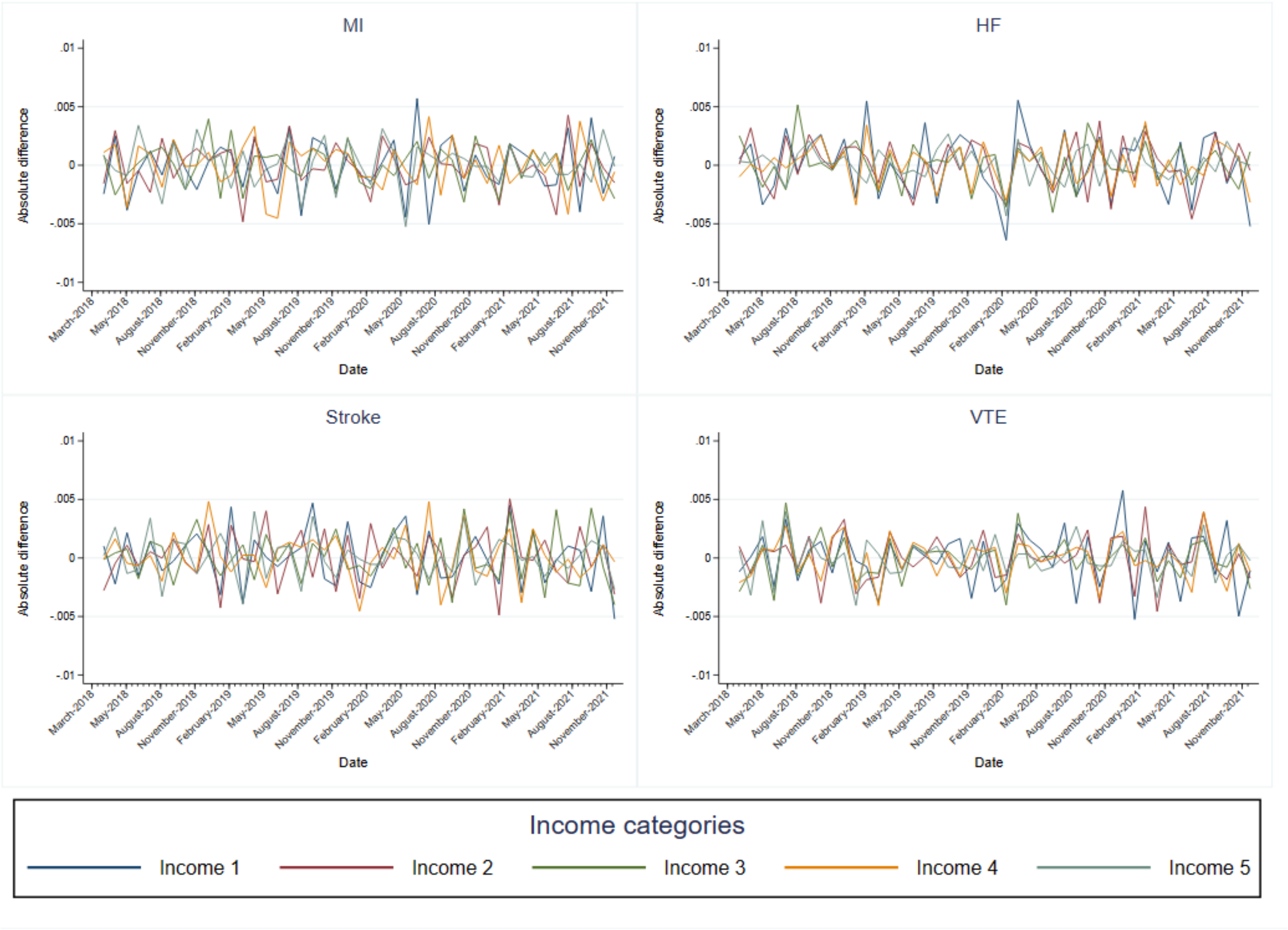

Supplement: online supplemental file 1 [file bmjopen-14-10-s001.pdf]
