# Supplementary material for: Impacts of the COVID-19 pandemic on deprivation-level differences in cardiovascular hospitalisations: a comparison of England and Denmark using the OpenSAFELY platform and National Registry Data
Source: BMJ Open. 2024 Oct 15;14(10):e088710. doi: 10.1136/bmjopen-2024-088710 (PMC11481132; doi:10.1136/bmjopen-2024-088710)
Supplement: Supplementary file 2 [file bmjopen-2024-088710.reviewer_comments.pdf]

PEER REVIEW HISTORY

BMJ Open publishes all reviews undertaken for accepted manuscripts. Reviewers are asked to complete a checklist review form and are provided with free text boxes to elaborate on their assessment. These free text comments are reproduced below.

ARTICLE DETAILS

Title (Provisional)

Impacts of the COVID-19 pandemic on deprivation-level differences in cardiovascular hospitalisations: A comparison of England and Denmark using the OpenSAFELY platform and National Registry Data

Authors

Costello, Ruth E; Pedersen, Lars; Henderson, Alasdair; Tazare, John; Sørensen, Henrik T. Toft; Vandenbroucke, J; Mansfield, Kathryn; Mahalingasivam, Viyaasan; Zheng, Bang; Carreira, Helena; Bidulka, Patrick; Piehlmaier, Dominik Manuel; Wong, Angel Yun Sum; Warren-Gash, Charlotte; Hayes, Joseph; Quint, Jennifer K; Katikireddi, Srinivasa Vittal; Mackenna, Brian; Mehrkar, Amir; Bacon, Sebastian; Goldacre, Ben; Tomlinson, Laurie A; Langan, Sinead; Mathur, Rohini; Collaborative, The LH&W NCS (or CONVALESCENCE); Consortium, The OpenSAFELY

VERSION 1 - REVIEW

**Reviewer**	**1**
**Name**	**Gharaibeh , Lobna**
**Affiliation**	**Al-Ahliyya Amman University, Biopharmaceutics and clinical pharmacy**
**Date**	**28-Jun-2024**
**COI**	**none**

Dear authors, thank you for your great work and effort. Please consider these comments:

Abstract:

1- The authors state in the abstract-results that they saw deprivation gradients and the wholes paragraph is about these differences but in the conclusion they write "we did not find that the pandemic substantially worsened------". Please re-write the either one so that the conclusion matches the result

Methods-ethical approval:

1- Please state the ethical approval you obtained from Denmark

Results

1- Statistics: Where are the p values in tables 2, 3A, and 3 B and the results for Poisson regression

Discussion: I suggest more emphasis on the impact of your findings

1- In the discussion there isn't any focus on possible causes on the differences found in hospitalization across deprivation quantiles and their implications on clinical practice or health policies. lines 18-43 in page 18-the last paragraph the authors compare the results but do not comments on the importance of their findings

2- The authors may also comment on the interaction between hospitalization patterns pre-post pandemic in the most deprivileged and the fact that most of people in this group are young

---

**Reviewer**	**2**
**Name**	**Sehgal, Anika**
**Affiliation**	**University of Calgary, community health sciences**
**Date**	**12-Jul-2024**
**COI**	**None.**

Individuals living in deprived areas are more likely to have CVD and have a higher risk of dying from CVD compared with those living in the least deprived areas (2–4) - change to living in lesser deprived areas.

Diversion of healthcare resources to pandemic management has negatively affected non-COVID-related healthcare provision, including prevention activities, potentially creating or worsening physical and mental health (9) - physical and mental health issues?

The negative impacts of the pandemic have been compounded by the rising cost-of-living crisis which may have further widened socioeconomic inequalities (10). - may have or has?

We do not know which aspects of the pandemic have been driving non-COVID health consequences. - what are non-COVID health consequences? which are you referring to here?

The UK experienced one of the worst COVID-19 outbreaks in Europe. Several Scandinavian countries have experienced better COVID-19 outcomes and faster healthcare system recovery (13). Confirmed COVID-19 deaths were higher in the UK compared to Denmark and the stringency of COVID-19 measures such as school closures, workplace closures and travel bans were higher in the UK compared to Denmark (14) supplementary materials. We do not know which aspects of the pandemic have been driving non-COVID health consequences. Comparing countries with different pandemic curves, where different measures were taken at different times may offer insights into which factors, if any, drive between-country differences, potentially informing policy for future infectious disease outbreaks. - this whole paragraph could be improved and expanded to better set the stage for the current paper.

Further to my last point, you state “We aimed to examine the impact of the pandemic on deprivation-related inequalities in hospitalisations for CVD conditions in Denmark and England between March 2018 and December 2021.” though no clear explanation is provided for why you chose Denmark and England? Furthermore why 2018-2021 when it is known that the strain put on health systems exceeded the ‘peak’ of COVID infections, whereby many healthcare systems today are still experiencing the impacts of the pandemic. In any case, this should be included as a limitation.

Hospital admissions with ICD-10 code for heart failure, MI, stroke or VTE, as the primary reason for admission (this refers to primary reason for spell in hospital) - this does not include people who may not have had their primary reason for admission to be something else, what about COVID positive people being admitted, their primary reason for admission would be COVID, regardless of if they were experiencing heart failure, MI, stroke or VTE.

VERSION 1 - AUTHOR RESPONSE

Response to reviewers

We thank the reviewers for their helpful comments and suggestions that have improved the paper. We have responded to each comment below.

Reviewer: 1

Dr. Lobna Gharaibeh , Al-Ahliyya Amman University

Comments to the Author:

Dear authors, thank you for your great work and effort. Please consider these comments:

Abstract:

1- The authors state in the abstract-results that they saw deprivation gradients and the wholes paragraph is about these differences but in the conclusion they write "we did not find that the pandemic substantially worsened------". Please re-write the either one so that the conclusion matches the result

Response: Thank you for this comment. We have added statements to the results to indicate where there were little differences (Abstract results, Page 2). The results now reads as follows (the additional text is italicised):

“Based on pre-pandemic trends, in England, there were an estimated 2608 fewer admissions than expected for heart failure in the most deprived quintile during the pandemic, compared to an estimated 979 fewer admissions in the least deprived quintile. For all other outcomes there was little variation by deprivation quintile. In Denmark, there were an estimated 1013 fewer admissions than expected over the pandemic for MI in the most deprived quintile, compared to 619 in the least deprived quintile. Similar trends were seen for stroke and VTE, though absolute numbers were smaller. Heart failure admissions were similar to pre-pandemic levels with little variation by deprivation quintile.”

Methods-ethical approval:

2- Please state the ethical approval you obtained from Denmark

Response: Apologies for this oversight. We have added the following details to the ethics statement, Page 24:

“ In Denmark, registry studies do not require ethical approval, but are reported to the Data Protection Board (Case number: 2016-051-000001/1793).”

Results

3. Statistics: Where are the p values in tables 2, 3A, and 3 B and the results for Poisson regression

Response: We have not provided P-values as it is not recommended to base interpretation of results on P-values (1). The STROBE checklist also does not recommend including P-values in descriptive tables, as P-values should not be used to determine whether a variable is a confounder (2). On a practical level, given the large sample size (n≈20 million), all p-values are highly significant (see also 95% CIs). Therefore, p-values do not provide readers with additional information beyond the reported results in the regression tables.

1. Amrhein V, Greenland S, McShane B. Scientists rise up against statistical significance. Nature. 2019 Mar;567(7748):305–7.

2. Vandenbroucke JP, Elm E von, Altman DG, Gøtzsche PC, Mulrow CD, Pocock SJ, et al. Strengthening the Reporting of Observational Studies in Epidemiology (STROBE): Explanation and Elaboration. PLOS Med. 2007 Oct 16;4(10):e297.

4. Discussion: I suggest more emphasis on the impact of your findings

Response: Thank you for this helpful suggestion, we have added the following sentence to the discussion (Page 20):

“This is important for future pandemic preparedness and understanding the optimal response that does not further inequalities.”

5. In the discussion there isn't any focus on possible causes on the differences found in hospitalization across deprivation quantiles and their implications on clinical practice or health policies.

Response: Thank you for this comment, we agree understanding the causes is important. We do discuss the different restrictions in the two countries in the Discussion, Page 19, and how this may have impacted cardiology services and explain some of the findings. We have updated the text to make this more explicit:

“There are potential explanations for this; the speed of response was quicker in Denmark, which resulted in less stringent restrictions in Denmark, compared to England (supplementary materials). There were fewer COVID-19 deaths in Denmark compared to England (26).”

In general we decided not to focus on possible causes as this work is descriptive. As noted in the limitations “since our results are descriptive, they help to generate hypotheses of potential mechanisms of differences observed, but they do not provide insight into the causes of any observed differences.”

6. lines 18-43 in page 18-the last paragraph the authors compare the results but do not comments on the importance of their findings

Response: We have added a sentence related to this, please see response to question 4 above.

7. The authors may also comment on the interaction between hospitalization patterns pre-post pandemic in the most deprivileged and the fact that most of people in this group are young

Response: Thank you for this suggestion, we have commented on the age profile of the English cohort in the discussion, Page 18:

“In both England and Denmark people in the most deprived quintile had a higher prevalence of diabetes and COPD; in England the mean age of people in the most deprived group was lower than for those in other deprivation quintiles.“

Reviewer: 2

Dr. Anika Sehgal, University of Calgary

Comments to the Author:

1. Individuals living in deprived areas are more likely to have CVD and have a higher risk of dying from CVD compared with those living in the least deprived areas (2–4) - change to living in lesser deprived areas.

Response: Thank you for this suggestion, the text has been updated as suggested as follows (Introduction, Page 4):

“ Individuals living in deprived areas are more likely to have CVD and have a higher risk of dying from CVD compared with those living in the less deprived areas (2–4).”

2. Diversion of healthcare resources to pandemic management has negatively affected non-COVID-related healthcare provision, including prevention activities, potentially creating or worsening physical and mental health (9) - physical and mental health issues?

Response: This sentence is referring to worsening health, including both physical and mental health, therefore we have updated this sentence as follows:

“Diversion of healthcare resources to pandemic management has negatively affected non-COVID-related healthcare provision, including prevention activities, potentially worsening physical and mental health (9)”

3. The negative impacts of the pandemic have been compounded by the rising cost-of-living crisis which may have further widened socioeconomic inequalities (10). - may have or has?

Response: We have clarified this and included an additional reference, introduction, Page 4:

“The negative impacts of the pandemic have been compounded by the rising cost-of-living crisis which has further widened socioeconomic inequalities (10,11).”

4. We do not know which aspects of the pandemic have been driving non-COVID health consequences. - what are non-COVID health consequences? which are you referring to here?

Response: Sorry if this was not clear. We intended this text to refer to any non-COVID health issues, this could include hospital admissions as we have studied, but could refer to any sort of ill health that is not related to COVID. After re-reading this paragraph we have taken out this sentence.

5. The UK experienced one of the worst COVID-19 outbreaks in Europe. Several Scandinavian countries have experienced better COVID-19 outcomes and faster healthcare system recovery (13). Confirmed COVID-19 deaths were higher in the UK compared to Denmark and the stringency of COVID-19 measures such as school closures, workplace closures and travel bans were higher in the UK compared to Denmark (14) supplementary materials. We do not know which aspects of the pandemic have been driving non-COVID health consequences. Comparing countries with different pandemic curves, where different measures were taken at different times may offer insights into which factors, if any, drive between-country differences, potentially informing policy for future infectious disease outbreaks. - this whole paragraph could be improved and expanded to better set the stage for the current paper.

Response: Thank you for this suggestion. This paragraph has been expanded with an additional sentence about the Danish restrictions. In addition, we have expanded the paragraph above to provide more background to the current study (Introduction, Page 4).

“The negative impacts of the pandemic have been compounded by the rising cost-of-living crisis which has further widened socioeconomic inequalities (10,11). During the early pandemic period, there were reports of fewer CVD admissions (7,12–14). One systematic review examining the impact of the COVID-19 pandemic on CVD related care (15) highlighted reduced and delayed CVD-related hospital admissions, except for cardiac arrests, and increased CVD mortality. In the UK, there were steeper drops in unscheduled hospital admissions in the most deprived, compared to the least deprived groups, though this was not specific to CVD admissions (7). However, a Swiss study of deprivation and CVD found that there were no changes in the relative patterning of inequalities resulting from the pandemic (16).

The UK experienced one of the worst COVID-19 outbreaks in Europe and some of the most severe outcomes from COVID-19 (17). In contrast, several Scandinavian countries have experienced better COVID-19 outcomes and faster healthcare system recovery (18). Denmark imposed strict restrictions earlier than the UK, and other countries (14). Although the UK imposed more stringent and longer lasting measures, confirmed COVID-19 deaths were higher in the UK compared to Denmark. This suggests that timeliness of intervention rather than duration, was of paramount importance in preventing COVID-19 mortality (19) (Figure S1, supplementary materials). Comparing inequalities in the indirect effects of the pandemic between countries with different pandemic curves, where different measures were taken at different times will be important for informing policy for future infectious disease outbreaks and ensuring that future mitigation measures do not exacerbate inequalities. ”

6. Further to my last point, you state “We aimed to examine the impact of the pandemic on deprivation-related inequalities in hospitalisations for CVD conditions in Denmark and England between March 2018 and December 2021.” though no clear explanation is provided for why you chose Denmark and England? Furthermore why 2018-2021 when it is known that the strain put on health systems exceeded the ‘peak’ of COVID infections, whereby many healthcare systems today are still experiencing the impacts of the pandemic. In any case, this should be included as a limitation.

Response: Thank you for this comment. We chose two different countries based on their differing healthcare systems and responses to the pandemic, we have elaborated in the paragraph (see Response 5 above).

In terms of the choice of study period, this was based on data availability, which was described in the Methods:

"In both settings, the study period was 1st March 2018 to 31st December 2021. This was to give 2 years of data prior to the start of the pandemic for comparison, the study ended on 31st December 2021 as Danish data were only available up until this date."

In addition we have acknowledged the study period dates in the Discussion, Page 20:

" Our study period ran until the end of 2021, longer than most previous studies (which largely ended in 2020) (33–35), allowing us to describe the longer-term impacts of the pandemic, although we acknowledge there could still be impacts later than 2021."

7. Hospital admissions with ICD-10 code for heart failure, MI, stroke or VTE, as the primary reason for admission (this refers to primary reason for spell in hospital) - this does not include people who may not have had their primary reason for admission to be something else, what about COVID positive people being admitted, their primary reason for admission would be COVID, regardless of if they were experiencing heart failure, MI, stroke or VTE.

Response: Thank you for this comment. We considered including people admitted to hospital with another diagnosis, who also experienced a cardiovascular event, but felt that this group did not match our study aim (to examine the impact of the pandemic on deprivation-related inequalities in hospitalisations for CVD conditions). Those admitted primarily for another reason could have complex reasons for experiencing a cardiovascular event, and results would have been difficult to interpret. Therefore we included only those with the specified cardiovascular primary reasons for admission.

Best wishes

Ruth Costello, on behalf of co-authors.

VERSION 2 - REVIEW

**Reviewer**	**2**
**Name**	**Sehgal, Anika**
**Affiliation**	**University of Calgary, community health sciences**
**Date**	**18-Sep-2024**
**COI**	

No further comments, thank you to the authors for making the changes based on my review.
